# Ideal Parent Figure method in the treatment of complex posttraumatic stress disorder related to childhood trauma: a pilot study

**DOI:** 10.1080/20008198.2017.1400879

**Published:** 2017-11-16

**Authors:** Federico Parra, Carol George, Khalid Kalalou, Dominique Januel

**Affiliations:** ^a^ Ville Evrard Clinical Research Unit, Nuilly-sur-Marne, France; ^b^ Psychology Department, Paris VIII University, Saint-Denis, France; ^c^ Ville Evrard Center of Psychotherapy and Psychotraumatology, Saint-Denis, France; ^d^ Psychology Department, Mills College, Oakland, CA, USA

**Keywords:** Adult attachment, trauma, AAP, Ideal Parent Figure method, CPTSD, DESNOS, incest, apego adulto, trauma, AAP, método de la Figura Parental Ideal, TEPTC, DESNOS, incesto, 童年依恋, 创伤, AAP, 理想父母形象方法, CPTS, DESNOS, 近亲结合, • CPTSD treatment requires a stabilization phase dedicated to decreasing symptoms and increasing psychological resources.• The Ideal Parent Figure (IPF) method treats attachment disturbances which tend to be present in CPTSD patients with childhood trauma.• In our sample of 17 adults with CPTSD, a short 4-session treatment using the IPF method significantly reduced symptoms and increased quality of life.• The results were stable eight months later.

## Abstract

**Background**: There is a consensus within the trauma field for the necessity of a three-phase treatment programme for complex posttraumatic stress disorder (CPTSD). This pilot study focuses on the stabilisation phase, the goal of which is the development of psychological resources and the reduction of disabling symptoms.

**Objective**: To test the efficacy of the Ideal Parent Figure (IPF) method as a stabilization treatment for CPTSD patients with a history of childhood trauma.

**Method**: The sample was comprised of 17 adults with a history of childhood trauma concomitant with CPTSD symptoms consulting at a clinic in France. Participants enrolled in a 5-week psychotherapy programme based on the IPF method, a semi-structured visualization programme designed to treat attachment disturbances. Measures of DESNOS symptoms, psychological symptoms, quality of life, and adult attachment were administered pre- and posttreatment as well as at 8-month follow-up.

**Results**: A significant decrease in symptom severity and attachment traumatization and a significant increase in quality of life were found, both with medium-to-large effect sizes. The 8-month follow-up assessment showed outcome stability.

**Conclusions**: These results suggest that treating attachment disturbances directly with an approach akin to the Ideal Parent Figure method may lead to fast and stable improvement for individuals with CPTSD.

## Abstract (es)

**Planteamiento**: Existe consenso dentro del campo del trauma sobre la necesidad de un programa de tratamiento de tres fases para el trastorno por estrés postraumático complejo (TEPTC). Este estudio piloto se centra en la fase de estabilización, cuyo objetivo es el desarrollo de recursos psicológicos y la reducción de síntomas incapacitantes.

**Objetivo**: evaluar la eficacia del método de la figura parental ideal (IPF, siglas en inglés de Ideal Parent Figure) como tratamiento de estabilización para pacientes con TEPTC con antecedentes de trauma infantil.

**Método**: La muestra estaba compuesta por 17 adultos con antecedentes de trauma infantil concomitante con síntomas de TEPTC que fueron a consulta en una clínica en Francia. Los participantes se inscribieron en un programa de psicoterapia de 5 semanas basado en el método IPF, un programa de visualización semiestructurado diseñado para tratar las alteraciones del apego. Se administraron mediciones de síntomas de DESNOS, síntomas psicológicos, calidad de vida y apego adulto antes y después del tratamiento, así como en un seguimiento a los 8 meses.

**Resultados**: Se encontró una disminución significativa en la gravedad de los síntomas y la traumatización por apego, y un aumento significativo en la calidad de vida, ambos con tamaños de efecto de mediano a grande. La evaluación de seguimiento a los 8 meses mostró estabilidad en los resultados.

**Conclusiones**: Estos resultados sugieren que el tratamiento de los trastornos de apego directamente con un enfoque similar al método de la figura parental ideal puede conducir a una mejoría rápida y estable para las personas con TEPTC.

## Abstract (zh)

**背景：**背景：治疗复杂创伤后应激障碍通用疗法包括三个步骤，这个试研究集中在稳定化阶段，目标是发展心理资源和减少受损症状。

**目标：**目标：考察理想父母形象法（IPF）对有童年创伤的CPTSD病人进行稳定化治疗的效果。

**方法：**方法：在一个法国诊所招募17名前来就医的成人，他们同时有童年创伤历史和CPTSD 症状，并登记参加了时长5周的基于IPF 的心理治疗项目。这个项目是一个半结构化的可视化项目，用于治疗依恋问题。在治疗前、治疗后和8个月后的追踪期，分别测量DESNOS 症状、心理症状、生活质量和成年依恋。

**结果：**结果：症状严重程度和依恋创伤显著减少，生活质量显著提高，两个效应都呈现中度到高度的效应量。8个月追踪期的测量显示了结果稳定性。

**结论：**结论：结果显示，在治疗依恋问题时直接使用类似理想父母形象疗法的方法可能给 CPTSD 患者带来快速和稳定的改善。

## 1. Introduction

### 1.1. Complex Posttraumatic Stress Disorder and attachment disturbances

Complex Posttraumatic Stress Disorder (CPTSD) is the psychiatric syndrome that best captures the constellation of symptoms associated with the developmental and repeated trauma experienced by survivors of childhood abuse (Herman, 1992; van der Kolk, Roth, Pelcovitz, Sunday, & Spinazzola, 2005). This syndrome is formally being included in the 11th version of the World Health Organization’s International Classification of Deceases (ICD-11) to be published in 2018 (Karatzias et al., 2017). CPTSD is understood in the literature as the concomitant presence of classic PTSD symptomatology in addition to Disorders of Extreme Stress Not Otherwise Specified (DESNOS) symptomatology, the latter involving alterations on six dimensions: (1) regulation of affect and impulses; (2) attention or consciousness (i.e. dissociative symptoms); (3) self-perception; (4) relations with others; (5) somatization; and (6) systems of meaning. The consensus among trauma experts is that CPTSD requires a three-phase treatment approach, as opposed to the single-phase approaches shown effective in the treatment of classic PTSD (Cloitre et al., 2012). The first phase of treatment is called *stabilization*. Central to this phase is a focus on the development of internal resources, often underdeveloped in this population, and on fostering the psychological competencies necessary to cope with both daily life stressors and the stress inherent to a later exposure treatment (Parnell, 2013). Reducing symptoms is also crucial to this stabilization phase (Cloitre et al., 2012). The second phase of treatment, often called *processing*, directly addresses the unresolved aspects of the patient’s memories of trauma so that they can be reintegrated into adaptive representations of self, relationships, and the world. Typically, this phase involves some form of exposure treatment (e.g. EMDR). In the third and last phase of treatment, *reconnection*, the focus is on facilitating the transition from treatment to greater engagement in relationships, work, education, and community life (Luxenberg, Spinazzola, van der Kolk, Hidalgo, & Hunt, 2001). Despite this clinical consensus about treatment, studies demonstrating treatment outcomes specifically for CPTSD are rare. A study by Dorrepaal et al. (2012) measured the outcomes of a 5-month stabilization (phase 1) treatment programme in a sample of 31 CPTSD patients that integrated cognitive-behavioural therapy and psycho-education. Their design included a control group consisting of 28 CPTSD participants enrolled in treatment as usual (TAU) for the same amount of time by a psychotherapist, psychiatric nurse, or psychiatrist, including medication. The study used the Structured Interview for Disorders of Extreme Stress (SIDES) to measure DESNOS intensity of symptoms. Both the treatment and control participants experienced a statistically significant decrease in DESNOS intensity of symptoms with very large (>1.20) effect size for the active group and large effect size (>.80) for the TAU control group.

The childhood trauma that spurs CPTSD is typically parent abuse or problems associated with parental physical or psychological abandonment (Herman, 1992). In attachment theory, trauma is conceived as failures for parent attachment figures to provide for children’s protection and safety and constitutes a severe form of ‘abdication of care’ that compromises children’s capacities for developmental and mental health, emotion regulation, self-integration, and capacity to maintain relationships (Bowlby, 1982; George & Solomon, 2008; Lyons-Ruth & Jacobvitz, 2016). Further, attachment theory posits that early interactions with parental attachment figures are internalized as representational models that contribute to evaluations of worth, and appraisals of self as competent and worthy of care later in life (Bowlby, 1982; Bretherton & Munholland, 2016). Representational models of self and attachment figures formed within the context of childhood trauma are dysregulated (Solomon & George, 2011). This means that, when invoked, these representational models induce a state in which behaviour and thought become disorganized and disoriented by either emotional flooding or attempts to prohibit or block emotions from consciousness (Bowlby, 1982; Solomon & George, 2011).

Research shows that adults who have not been able to ‘resolve’, that is re-organize mental representations (Bowlby, 1982) from experiences of childhood trauma are over-represented in psychiatric populations (Stovall-McClough & Dozier, 2016). Childhood traumatic interactions therefore impede the development of regulation strategies that buffer adults from severe psychological distress, and can compound it (Bacon & Richardson, 2001; Ma, 2006; Sroufe, Coffino, & Carlson, 2010). In addition to this psychological burden, survivors of prolonged childhood trauma often experience persistent physical health difficulties, including in their digestive, cardiopulmonary, and urogenital systems (Luxenberg et al., 2001).

Representational models of attachment can be stable or change, depending on life circumstances (Waters, Hamilton, & Weinfield, 2000). With regard to trauma, the nature of change depends on whether later experiences are supportive or threatening (Cyr, Euser, Bakermans-Kranenburg, & Van IJzendoorn, 2010). Previous studies have shown that the visualization of imaginary responsive attachment figures can successfully elicit a temporary feeling of ‘security’ (i.e. free of fear; Bowlby, 1982) that is associated with increasing psychological resources; this in turn supports coping with adversity (Mikulincer, Hirschberger, Nachmias, & Gillath, 2001; Mikulincer & Shaver, 2007). A few therapeutic methods have been developed that take advantage of this principle with the goal of a more permanent change (Hammond, 1990; Parnell, 2013). In 2016, Brown and Elliott published a manual presenting a comprehensive treatment model for attachment disturbances, developed by the authors and a number of collaborators over the course of two decades. This manual described in detail the Ideal Parent Figure (IPF) method, a psychotherapeutic intervention designed specifically to help patients develop new, positive representational models of attachment. Their book includes the results of a pilot outcome study conducted by the authors using their treatment methodology with 12 participants in long-term psychotherapy (*M* = 3.4 years). All patients achieved an ‘earned secure’ attachment classification as measured by the Adult Attachment Interview (AAI; Findings by George C, Kaplan N, and Main M, unpublished data, 1985) towards the end of treatment, showing also a significant (*p* < .001) and very large (Cohen’s *d = *6.23) increase in coherence of mind, which is seen in the field as the single best continuous index of attachment security (Brown & Elliott, 2016).

### 1.2. Using the Ideal Parent Figure method to treat CPTSD

The psychotherapy programme implemented in this study is based on the IPF method (Brown & Elliott, 2016), which uses guided imagery to help participants change their attachment representations. During our sessions, participants were guided by the therapist to vividly imagine themselves as children, and then to interact with a set of new parents, not the parents or any caregivers they grew up with. The therapist guidance included descriptions of specific qualities that these imagined parents possess: they are protective of their child, capable of soothing them, attuned to the child’s emotional states, capable of fostering their self-development through encouragement for inner and outer exploration, and expressive about their delight in the very presence of the child. These specific parent figure qualities are based on attachment needs that, when met, are considered to promote secure attachment (Ainsworth, Blehar, Waters, & Wall, 1978; Brown & Elliott, 2016). The therapist instructions encouraged the participants to either imagine being with parent figures who were entirely constructed from their imagination, or to imagine parents who were loosely based on fictional or real characters that were experienced by the participant to possess the aforementioned qualities. The participants were instructed to truly immerse themselves in the visualization, experiencing physical sensations in accordance to what is being imagined, like when we dream. Importantly, when needed, the therapist helped participants to differentiate imagination from memory, making sure participants were not simply recalling their own past experiences during the guided imagery. In short, the purpose was to co-create new experiences.

In some cases, the differences between imagination and memory were not obvious for a participant. Other times a participant felt that they could not imagine something never experienced before. Sometimes a participant would have trouble embodying the visualization. In all three cases, the therapist had the participant imagine something never experienced before and that was prone to induce physical sensations (e.g. climbing the Himalayas), to bring forth a couple of insights: imagination is not tied to prior experience and, when something is imagined intensely enough, the body starts reacting and sensations are felt, just like during vivid dreams.

The visualization was organized as a series of five semi-structured scenes; each scene corresponded to a specific attachment-need/parent-quality pair. For example, one scene was about a situation in which the participant-as-a-child felt emotionally overwhelmed (attachment need is to be soothed), and how the imagined parents promptly responded by comforting and soothing the child (parent quality is sensitivity to the child’s need). The specific details about *what* exactly overwhelmed the child and of *how* exactly the parent figures met that need were left for the participant to fill in. The visualization was preceded by a short therapist-led body-scan relaxation exercise. After going through the scenes, participants were instructed to internally review their experience of the visualization, integrating positive emotions experienced throughout.

### 1.3. The current study

This pilot study is the first independent empirical examination of the IPF method, and the first to empirically investigate IPF effects on CPTSD participants with a history of childhood trauma. The study took place between March 2015 and February 2017 at the Ville Evrard Center of Psychotherapy and Psychotraumatology, a government-funded public psychotherapy centre located in Saint Denis, France. The pilot study was run in collaboration with the Ville Evrard Clinical Research Unit and Paris VIII University. Our trial was open and uncontrolled.

We hypothesized that our treatment programme would decrease DESNOS symptoms, psychological symptoms in general, and attachment dysregulation related to childhood trauma. We hypothesized that treatment would increase quality of life.

## 2. Method

### 2.1. Participants

Participants were referred to our programme by a psychiatrist member of our team. After a routine in-take clinical interview administered at our psychotherapy centre, individuals presenting symptoms of PTSD and DESNOS, and reporting histories of childhood trauma, were encouraged to join our treatment programme. Our sample was comprised of 18 participants (14 female; age *M *= 42 years, *SD *= 13.36). Seventeen participants completed the study, assisting all sessions.

Eleven participants had one or more formal DSM diagnoses at the time of inclusion. Seven participants were taking psychiatric medications during the course of treatment, including sleeping pills, antidepressants, and mood stabilizers. Three participants were concomitantly seeing a psychodynamic psychotherapist. All of these parallel treatments were stable (> 3 months) at the time our treatment programme began. Study inclusion was based on the following criteria:

- PTSD diagnosis by a psychiatrist of the centre
- A score beyond the clinical cut-off in at least one kind of childhood trauma in the Children Trauma Questionnaire (CTQ)
- A DESNOS severity of symptoms score superior to 33.62 ^1^
- French language fluency
- Being 18 years or older

The study was approved by Paris VIII University. Written informed consent was obtained from all participants by our staff before starting the programme.

### 2.2. Treatment programme

The treatment was offered at no charge (consistent with the French public mental health care system) and consisted of four weekly therapy sessions using the IPF method previously described. Patients went through the visualization once per session.

Based on their clinical experience, Brown and Elliott (2016) encourage individualizing IPF imagery for each patient according to the patient’s attachment-specific needs, and they warn that the use of a ‘one-size-fits-all’ IPF script could decrease the effectiveness of the intervention. The present work used a unique script despite that advice, a research design meant to improve the validity of our results by reducing confounding factors in the analysis of treatment outcomes.

Using this script, our visualization lasted about 16 minutes. A member of our team that had trained in the IPF method was in charge of the therapy sessions. A recording of the visualization script was given to all participants towards the end of the first session and they were encouraged to repeat the visualization practice at home by listening to the recording as many times as they wanted between sessions.

### 2.3. Measures

*Childhood Trauma Questionnaire* (CTQ; Bernstein, Ahluvalia, Pogge, & Handelsman, 1997). The CTQ is a self-report instrument composed of 28 items that assess different forms of childhood trauma: emotional, physical, and sexual abuse, as well as experiences of emotional and physical neglect. Responses are made on a 5-point Likert scale from 1 (Not true) to 5 (Very often true). Clinical cut-off scores are provided by the authors for each kind of trauma. The French translation of the instrument was developed by Paquette, Laporte, Bigras, and Zoccolillo (2004), without back-translation procedures. Validity and reliability for the French translation was tested on 394 subjects (71% female), recruited from randomly chosen and diverse institutions in Montreal. Internal consistency of the scales evaluated by Cronbach’s alphas was excellent varying between 0.68 and 0.91. Test-retest reliability of the scales was evaluated using Pearson correlation with 12 subjects and was excellent, varying from 0.73 to 0.94. Concurrent validity was measured against the Self-Report Family Inventory (SFI; Beavers, Hampson, & Hulgus, 1990). All five scales of the CTQ were correlated, as expected, with those of SFI. CTQ scores were negatively correlated with family health, family cohesion, and expression of emotions, and positively correlated to family conflicts (Pearson *r* ranged from 0.35 to 0.82). Five experts in child abuse were consulted to provide cut-off scores for each of the five scales; the means of all experts’ cut-off scores were used. A factor analysis forced to five factors explained 55% of the sample’s variance replicating the English counterpart.

*Self-Report Inventory for Disorders of Extreme Stress* (SIDES-SR; Pelcovitz et al., 1997). The SIDES-SR is a self-report questionnaire comprised of 45 items developed as a diagnostic tool to evaluate the presence of DESNOS as well as the severity of its symptoms. SIDES-SR assesses the presence of symptoms in the respondent using a dichotomous scale (yes/no) and the intensity of symptoms using a 4-point Likert scale. In this study we used the severity of symptoms score of SIDES-SR as a measure of overall DESNOS intensity. Payer (2012) translated the instrument to French, using Vallerand’s (1989) back-translation methodology. Payer (2012) conducted a validation study with 438 francophone Canadian adults (243 students, 196 from local clinics, 79% female). Payer first compared her translation to the English original through a Principal Axis Factor analysis through which she found the same five factors revealed in the English version by Scoboria, Ford, Lin, and Frisman (2008). Payer stablished the instrument’s internal validity using Cronbach’s alpha, finding a good coefficient of .82. Test-retest reliability was tested on a subsample of 82 adults, finding a high correlation (*r *= .79) between tests. Convergent, discriminant, and concurrent validity were also confirmed against the Modified PTSD Symptom Scale - Self-Report (Falsetti, Resnick, Resick, & Kilpatrick, 1993), the Short Symptom Inventory (Derogatis, 1977), the Schema questionnaire (Young & Brown, 1994), and the Early Trauma Inventory (Bremner, Bolus, & Mayer, 2007). Worth mentioning, the measure was found to be able to distinguish well individuals that suffered childhood trauma (Payer, 2012).

*Brief Symptom Inventory* (BSI; Derogatis, 1993). An abbreviated version of the well-known Symptom Checklist 90-R, the BSI is a self-report questionnaire comprised of 53 items answered using a Likert 5-point scale. The BSI evaluates nine clinically significant psychological symptom patterns: somatization, obsession compulsion, interpersonal sensitivity, depression, anxiety, hostility, phobic anxiety, paranoid ideation, and psychotic symptoms. These nine scores are summed to produce a Global Severity Index (GSI), a measure of the intensity of psychological symptoms that is the most frequently used BSI’s indicator in psychotherapy evaluations (Ryan, 2007). The BSI was validated originally with three different samples: a sample of 1002 heterogeneous psychiatric out-patients; a sample of 719 non-patient normal subjects; and a sample of 313 psychiatric in-patients (Derogatis & Melisaratos, 1983). The GSI score demonstrated strong internal validity with a Cronbach’s alpha coefficient of .97 as well as high test-retest reliability (*r* = .87; Derogatis, 1993). Derogatis (1993) also showed acceptable internal validity for the nine dimensions using Cronbach’s alphas, within the range of .71 to .85, as well as high test-retest reliability with Pearson correlations within the .68 to .91 range. In terms of concurrent validity, correlations between BSI and other psychometric instruments evaluating similar symptoms were moderate to strong. The French version of the BSI used in our study was translated by the Association nationale pour le développement de la qualité dans les hôpitaux et les cliniques (ANQ; 2012, version 2) using a back-translation process conducted by experts native in English and French.

*World Health Organization Quality of Life BREF* (WHOQOL-BREF; Harper, 1996). An abbreviated version of the well-known World Health Organization Quality of Life 100 (WHOQOL-100), the WHOQOL-BREF is a self-report questionnaire consisting of 26 items elaborated by the WHOQOL team. The responses are given using a Likert 5-point scale. It evaluates four domains of quality of life and can generate a general quality of life score, which was used in this study. During a field trial validation study with 4104 participants from different centres worldwide, the WHOQOL team found strong correlations between the scores in the domains within WHOQOL-100 and WHOQOL-BREF, in the range .89–.95 depending on the specific domain. The Cronbach’s alpha coefficients for each of the domains were within the .66–.84 range. The abbreviated version was also comparable to the original test in its capacity to discriminate groups of healthy vs. unhealthy subjects (Harper, 1996). The French translation used in this study was performed and validated by the WHOQOL group using their complex back-translation methodology during the field trials (World Health Organization, 1998).

*Adult Attachment Projective Picture System* (AAP; George & West, 2012). The AAP is a free-response representational assessment of attachment in adults. Attachment classification is derived from the response patterns to seven standardized attachment scenes that portray individuals alone and in potential attachment dyads. The instrument’s validity was established in a study comprised of 144 participants (69% female) represented by two subsamples, one from Calgary, Alberta, Canada (*n* = 73) and the other from northern California (*n* = 71) (George & West, 2012). In terms of convergent validity, AAP classifications were compared to AAI classifications which are considered gold standard (Ravitz, Maunder, Hunter, Sthankiya, & Lancee, 2010). George and West (2012) reported on a range of validity statistics. The comparison was made using the Kappa statistic, showing high agreement: Kappa = .84, *p* < .000. Sixty-nine participants took the AAP a second time 12 weeks later to investigate test-retest reliability, which was found to be high (Kappa = .78, *p* < .000). Inter-rater reliability in the AAP was measured by comparing three different raters: Kappa = .85, *p* < .000 was found between rater 1 and 2, and Kappa = .79, *p* < .000 between rater 1 and 3. Discriminant validity tests showed that AAP classifications were not influenced by verbal intelligence and social desirability (George & West, 2012). The AAP was validated for francophone populations in Quebec by Béliveau and Moss in 2005 on a sample of 123 mothers; their validation study included measures of stressful life events, parenting stress, helplessness, depression, socio-economic, and global psychosocial functioning to test discriminant and convergent validity, and demonstrated that the AAP was independent of measures of socio-economic and more global psychosocial functioning. In our study, transcripts of the AAP responses were classified by a member of our team, blind to all information about the participants. Inter-rater reliability was obtained for 10 cases that were classified by two external AAP master judges, with 80% and 90% of agreement respectively. In addition to classification group, the AAP provides a continuous score that is derived by summing the frequency of the so-called Segregated Systems Trauma (SStr) markers in the transcript (Buchheim & George, 2011). This continuous score measures the intensity of attachment dysregulation related to childhood trauma (Buchheim & George, 2011) and was used in the present study as an attachment outcome measure.

### 2.4. Procedure

Study measures were administrated one week prior to the beginning of treatment, one week after the end of the programme, and at follow-up approximately eight months (*M *= 8, *SD *= 2) after the end of the programme. Self-report measures were completed in the waiting room of the psychotherapy centre. The AAP interview was administered by a trained member of our team.

### 2.5. Statistical analyses

Due to small sample size (*n* = 17), we first conducted Kolmogorov-Smirnov and Shapiro–Wilk tests and examined histograms and Q-Q plots for each of our variables to verify normality of the distributions. These tests demonstrated that the data was normally distributed.

Statistical analyses were therefore performed using paired *t*-tests.^2^ All analyses were conducted using IBM SPSS Statistics Version 23. Cohen’s *d* was calculated for all pairs using the variance of the first of the two compared groups. The results of post hoc power analyses for appropriate sample size estimation are included for each measure. A priori power analyses could not be conducted because there is no prior published data about this therapeutic method on which to base appropriate effect sizes. Comparing treatment outcome between males and females was not possible given the low number of male participants that completed the study (*n* = 3).

## 3. Results

### 3.1. Childhood trauma and symptom severity at inclusion

In terms of childhood trauma, of the five types measured by the CTQ six participants experienced three types, three participants experienced four types, and one participant experienced all the five types. Eleven participants reported a history of childhood *sexual* abuse, including incest. The sample’s mean experienced childhood traumas was 2.88 (*SD *= .96). The mean SIDES-SR severity of DESNOS symptoms of our sample at inclusion was 61.18 (*SD *= 16.99).

### 3.2. Pretreatment (T1) to posttreatment (T2)

There was a significant decrease in symptoms scores and increase in quality of life scores from pretreatment to posttreatment, with medium-to-large effect sizes (*N* = 17).

**Figure 1.**
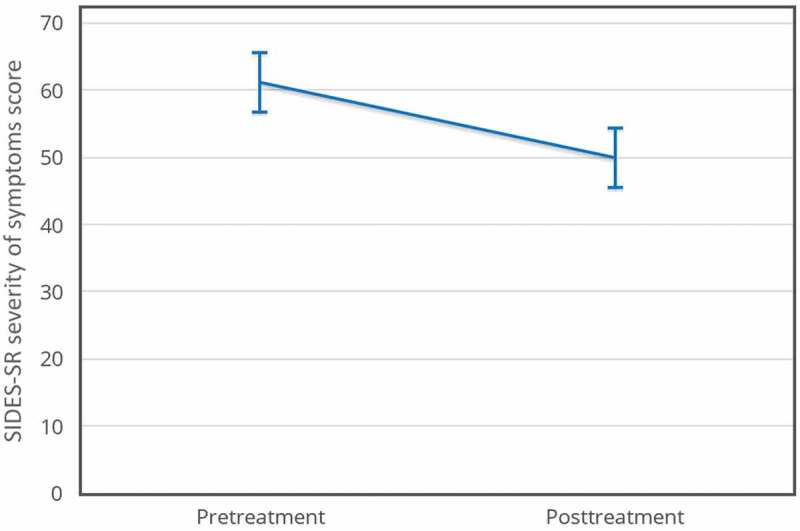
SIDES-SR severity of symptoms, *N* = 17.

SIDES-SR severity of DESNOS symptoms decreased (pretreatment *M *= 61.18, *SD *= 16.99; posttreatment *M *= 49.94, *SD *= 26.27) *t*(16) = 2.69, *p *< .05, *d* = 0.66. The effect size for this analysis exceeded Cohen’s (1988) convention for a medium effect (*d* = 0.50). The 95% confidence interval for the mean difference was 2.38 to 20.09. Using this effect size, a post hoc power analysis, demonstrated that a minimum of 21 participants should be recruited to achieve 0.8 power with a significance level (α) of 0.05 (see Figure 1).

**Figure 2.**
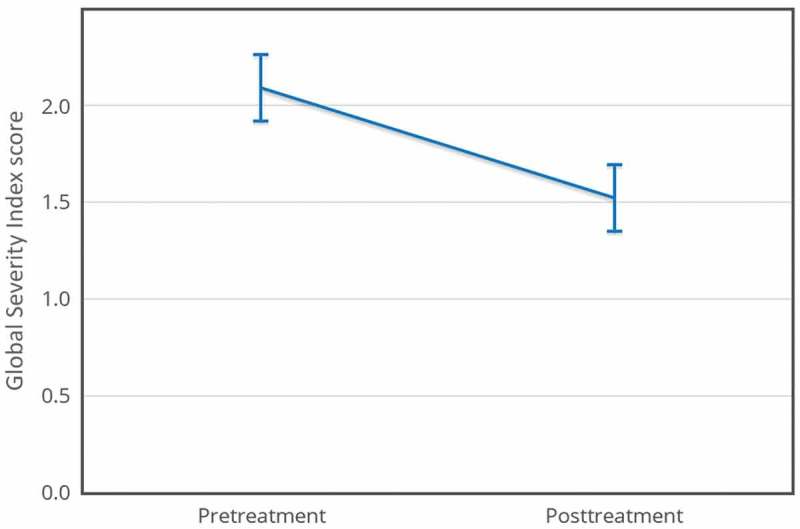
BSI Global Severity Index pretreatment/posttreatment, *N* = 17.

BSI GSI scores significantly decreased between pretreatment and posttreatment (pretreatment *M* = 2.09, *SD* = 0.59; posttreatment *M *= 1.52, *SD *= 0.94), *t*(16) = 3.52, *p *< .001, *d* = 0.97. This effect size exceeded Cohen’s (1988) convention for a large effect (*d* = 0.80). The 95% confidence interval for the mean difference was 0.23 to 0.91. Using this effect size, a post hoc power analysis revealed that a minimum of 15 participants should be recruited to achieve 0.8 power with a significance level (α) of 0.05 (see Figure 2).

**Figure 3.**
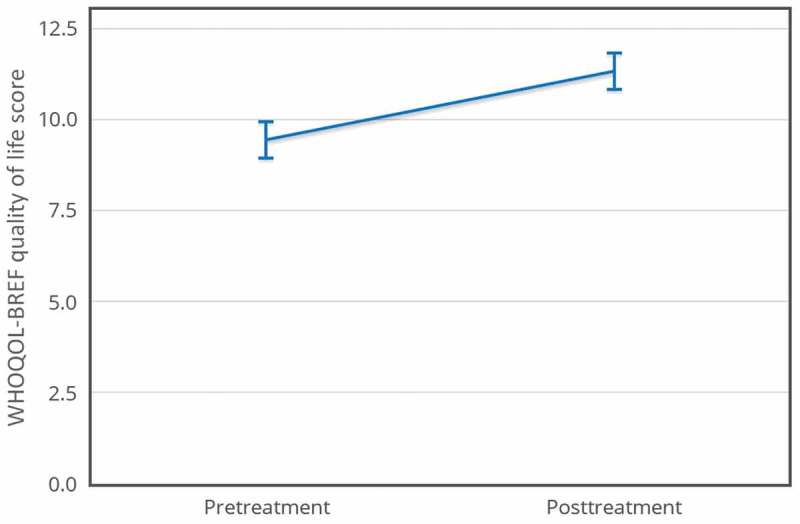
WHOQOL-BREF quality of life, *N* = 17.

WHOQOL-BREF quality of life scores increased (pretreatment *M *= 9.43, *SD *= 2.36; posttreatment *M *= 11.32, *SD *= 3.08), *t*(16) = −4, *p* < .001, *d* = 0.80. This effect size reached the level of Cohen’s (1988) convention for a large effect (*d* = 0.80). The 95% confidence interval for the mean difference was −2.88 to −0.89. Using this effect size, a post hoc power analysis revealed that a minimum of 15 participants should be recruited to achieve 0.8 power with a significance level (α) of 0.05 (see Figure 3).

**Figure 4.**
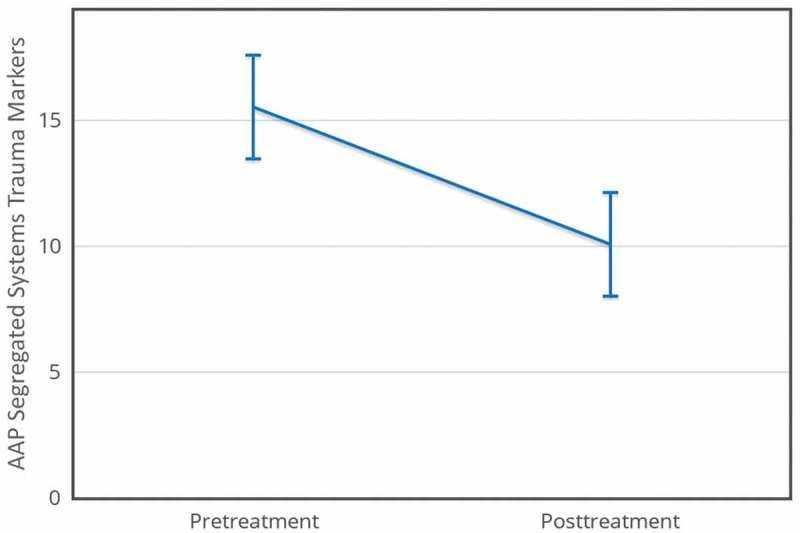
AAP Segregated Systems Trauma Markers, *N* = 15.

Analysis of treatment effects for traumatic dysregulation (SStr) was performed on 15 of the 17 cases (one pretreatment transcript was lost due to audio technical problems; one posttreatment interview was cancelled by the participant without the possibility to reschedule). There was a significant decrease in SStr frequency (pretreatment *M *= 15.53, *SD *= 9.05; posttreatment *M *= 10.07, *SD *= 6.09), *t*(14) = 2.84, *p* < .01, *d* = 0.60. This effect size exceeded Cohen’s (1988) convention for a medium effect (*d* = 0.50). The 95% confidence interval for the mean difference was 1.34 to 9.59. Using this effect size, a post hoc power analysis revealed that a minimum of 24 participants should be recruited to achieve 0.8 power with a significance level (α) of 0.05 (see Figure 4).

Figures 1–4 show means for all four measures pretreatment and posttreatment. The error bars are 95% within-subject confidence intervals (Loftus & Masson, 1994).

### 3.3. Posttreatment (T2) to follow up (T3)

A subsample of 13 participants was available for follow-up assessment for the three self-report measures approximately eight months (*M *= 8, *SD *= 2.2) after the end of the programme. Neither paired *t*-tests nor Wilcoxon Signed-Ranks tests between posttreatment and follow-up assessments showed any significant differences at follow-up for any of the three self-report measures. A hypothetical explanation was that the reduced size of the subsample used in the follow-up calculations (*n* = 13) could have produced a loss in statistical power, perhaps inducing type 2 errors. According to our own post hoc power analyses, a minimum of 21, 15, and 15 participants were required to achieve sufficient statistical power (0.8) to correctly reject the null hypothesis with regards to SIDES-SR, BSI GSI, and WHOQOL-BREF measures respectively with a significance level (α) of 0.05.

To test the fitness of this explanation, we repeated the pretreatment vs. posttreatment analysis for the SIDES-SR, BSI, and WHOQOL-BREF using the same *n* = 13 subsample (see Table 1).

**Table 1.** *t*-tests and Wilcoxon Signed-Ranks tests comparing pretreatment (T1) and posttreatment (T2) and posttreatment (T2) and follow-up (T3) scores for the follow-up subsample (*n* = 13).

	*T1–T2*			*T2–T3*		
	*t-test*		*Wilcoxon Signed-Ranks*	*t-test*		*Wilcoxon Signed-Ranks*
	*p*-value	effect size (Cohen’s *d*)	*p*-value	*p*-value	effect size (Cohen’s *d*)	*p*-value
SIDES-SR	0.09	0.51 (medium)	0.05	0.41	0.12 (very small)	0.39
BSI GSI	0.03	0.78 (medium)	0.03	0.57	0.08 (very small)	0.51
WHOQOL-BREF	0.01	0.68 (medium)	0.01	0.16	0.17 (very small)	0.25

Significant difference found between T1 and T2 for BSI and WHOQOL-BREF, in both tests. Significant difference found for SIDES-SR using Wilcoxon Signed-Rank test. No significant difference found between T2 and T3. SIDES-SR = Self-Report Inventory for Disorders of Extreme Stress; BSI GSI = Brief Symptom Inventory Global Severity Index; WHOQOL-BREF = World Health Organization Quality of Life BREF.

We found that a significant difference was still evidenced between pretreatment and posttreatment for BSI and WHOQOL-BREF, demonstrated using both parametric and non-parametric tests, whereas a significant difference was still shown for SIDES-SR using the Wilcoxon Signed-Rank test. Cohen’s *d* was still medium-to-large in the pretreatment vs. posttreatment comparison for the three measures in this subsample.

In contrast, posttreatment vs. follow-up parametric and non-parametric tests performed on the subsample were non-significant for SIDES-SR, BSI and WHOQOL-BREF, and Cohen’s *d* was in the very small range (< .20) for all three. These findings do not support our lack-of-power hypothetical explanation. We propose an alternative explanation in the discussion below.

### 3.4. Post hoc analyses

A large Pearson correlation (*r* = .60, *n* = 17, *p* = .01) was found between CTQ reported childhood trauma scores and SIDES-SR DESNOS reported severity of symptoms scores at inclusion, replicating findings by Payer (2012) about SIDES-SR concurrent validity. Analyses of the six dimensions of DESNOS that are individually measured by the SIDES-SR were conducted. Pretreatment vs. posttreatment comparisons using *t*-tests showed statistically significant changes with medium effect sizes for three of the six dimensions: regulation of affect and impulses, relationship with others, and systems of meaning. A trend (*p* < .10) with medium effect size was found for self-perception. No significant changes were found for either attention or consciousness (i.e. dissociative symptoms) or somatization (see Table 2).

## 4. Discussion

The current study focused on the outcomes of treating a group of severely traumatized participants with CPTSD symptomatology and a history of childhood trauma, employing a relatively novel technique designed specifically to treat attachment disturbances. We emphasize here the severity of DESNOS symptomatology in our participants at inclusion, which was almost double the mean reported in the validation study included in the SIDES scoring manual (Spinazzola, 2011).

Analyses confirmed our hypotheses. We found statistically significant improvement with medium-to-large effect sizes indicating a decrease in DESNOS symptomatology, psychological symptoms in general, and traumatic dysregulation, as well as an increase in quality of life following the completion of a very short treatment programme (*M *= 4.61 weeks, *SD *= 1.40). Follow-up for a sub-sample of 13 participants (76%) showed no significant changes in scores at 8-months following the end of treatment, and we suggest these findings be interpreted as the stability of the posttreatment results over time.

In comparison to the results of Dorrepaal et al. (2012), the difference between pretreatment and posttreatment for DESNOS severity of symptoms during our 1½-month-long programme produced a medium effect size (> .50) and was not as strong as the very large (> 1.20) effect size reported by them. Our treatment group, however, had more severe pretreatment symptoms than reported in the Dorrepaal et al. study, and our treatment period was four times shorter.

In order to explore these findings further, we performed post hoc analyses on the six dimensions of DESNOS that are individually measured by the SIDES-SR measure, and we found statistically significant changes with medium effect sizes for three of the six dimensions and a trend for another dimension. No significant changes were found for either attention or consciousness (i.e. dissociative symptoms) or somatization, suggesting that these aspects of the syndrome are not addressed by this treatment method and might require a different approach.

The design of the present study did not allow us to determine the mechanisms of change. However, our overall findings could be theoretically explained through the lenses of attachment theory. Specifically, IPF seems to reduce representation of self and relationships as threatening and traumatic; this reduction is indicated by the observed reduction of SStr marker frequency in the AAP. We suggest that the IPF method therefore could be seen as a mechanism of change, helping to change representational models through the visualization of more ideal caregiving relationships than those experienced during the participants’ own childhoods. We suggest that these new representational models were more efficient in helping to keep the participants regulated and restoring regulation when distressed. These processes would then be translated into a pronounced reduction of reported psychological symptoms and evaluations of having a higher quality of life than that which was reported before treatment.

We propose that the use at home of the recorded visualization was an instrumental factor contributing to these changes and observed outcomes; using the recording between formal sessions facilitated a daily experience (i.e. immersion) with positive caregiving experiences. Several participants indicated at follow-up that they were still using the recording at home as a coping mechanism to regulate distressing emotions after difficult life events. Although we did not keep a record of the exact amount of times participants used the recording at home between sessions, we anecdotally state here that one person used the recording a total of 52 times during the course of treatment.

The current study was the first independent empirical evaluation of the IPF method. The results suggest that this approach to treatment of attachment disturbances is poised as a promising approach to the treatment of CPTSD involving childhood trauma, particularly during the stabilization phase of treatment. This phase focuses on developing essential psychological competencies, such as emotional regulation, and on reducing disabling symptoms (Cloitre et al., 2012). The outcome of our IPF-based protocol is that regulation of affect and impulses, relationship with others (including trust in others), and systems of meaning (including anticipative beliefs about the world) were all improved, whereas disabling symptoms were reduced, meeting the stabilization goals.

During the conception of the present study, no hypotheses were made regarding possible changes in attachment classification. It seemed inappropriate to expect any change in overall mental representation of attachment as an outcome of such a short treatment programme. Post hoc analyses, however, revealed that some participants’ classifications did change (see Table 2). Specifically, out of the 15 participants for which AAPs were coded, four participants resolved their unresolved status during the course of treatment, and three participants that began treatment as insecure changed to secure. One case, however, changed from dismissing to unresolved, a negative outcome at face value. We hypothesize that such change is likely due to unravelling of defended trauma during treatment. These post hoc results, overall positive, are marked considering the short length of treatment, the gravity of the sample’s trauma histories, and the high test-retest reliability of the AAP instrument. They seem to replicate findings in Brown and Elliott (2016) own pilot study.

**Table 2.** AAP attachment classification.

Patient	T1	T2
1	Unresolved	Unresolved
2	Unresolved	Secure
3	Unresolved	Unresolved
4	Dismissing	Secure
5	Unresolved	Unresolved
6	Unresolved	Unresolved
7	Unresolved	Preoccupied
8	Dismissing	Dismissing
9	Dismissing	Unresolved
10	Unresolved	Secure
11	Unresolved	Unresolved
12	Unresolved	Dismissing
13	Unresolved	Unresolved
14	Unresolved	Unresolved
15	Unresolved	Unresolved

AAP = Adult Attachment Projective Picture System; T1 = pretreatment; T2 = posttreatment.

### 4.1. Limitations

As a pilot study, this work presents several limitations. As an open, uncontrolled trial, the study was not able to control for confounding factors such as the placebo effect, regression to the mean, or the effects of the therapeutic relationship. Though all concurrent psychotherapy and medication use was stable (> 3 months) at the time our intervention begun, this doesn’t completely rule out the risk of confounding effects.

Another limitation was the very short duration of our treatment programme. The IPF method was conceived as a longer-term therapy, typically lasting from several months to several years (Brown & Elliott, 2016). Thus, future studies should evaluate potential additional improvements derived from a longer continuous IPF treatment on CPTSD patients. From a cost-effectiveness perspective, however, the short length of our treatment protocol is a strength.

An additional limitation to the design of the current study was the absence of measures for classic PTSD symptomatology, such as the PCL (PTSD checklist) or the Trauma Screening Questionnaire (TSQ). This prevents determining whether classic PTSD symptoms changed over the course of treatment. Given the importance of PTSD symptoms to the CPTSD syndrome, this remains an empirical question for future studies to address.

Another limitation of the study is that we did not keep records of the number of times participants used the recording at home. Because of this, we cannot establish dose effects or provide guidelines about frequency of utilization for future treatment programmes.

Lastly, our post hoc power analyses demonstrated that our sample was too small for at least some of our analyses (e.g. SIDES-SR pretreatment to posttreatment). Correctly understood, however, this represents a strength rather than a weakness: the fact that medium-to-large effect sizes were detected in a small sample such as ours attest to the robustness of the findings.

### 4.2. Future directions

The therapist took notes during each of the treatment sessions for seven of the 17 participants. These notes contain both verbatim comments from the participants as well as clinical observations. This rich clinical material captures, perhaps better than statistics alone can, the profound transformation occurring for these participants throughout treatment. It is our intention to publish them in the future to complement the present work, together with qualitative analyses of participants’ experiences.

Other future steps include a more controlled research design with a larger sample. In particular, we think future studies would benefit from including measures specifically designed to test some of DESNOS dimensions, for example including the Difficulties in Emotion Regulation Scale (DERS; Gratz & Roemer, 2004) as a measure specifically designed to assess emotion regulation would allow for a more in-depth assessment of how much this dimension is impacted by treatment. Following Brown and Elliott (2016) guidelines, a future study could use pretreatment attachment test results to customize the IPF protocol to suit the patient’s specific attachment needs instead of relying on a one-size-fits-all script. Researchers might also explore outcome differences using a control group who received a ‘sham’ IPF protocol. Such protocol would use visualized scenes in which positive interactions happen with imagined parents, but in which said interactions are not attachment-related (e.g. ideal parents and the child go shopping). Such a design would allow for a fine-grained differentiation among confounding factors, as both groups would be subjected to almost the same exact treatment methodology.

Finally, future outcome studies on CPTSD treatment would benefit from adopting the new ICD-11 CPTSD nosography, which allows for a precise differentiation between CPTSD and classic PTSD.

### 4.3. Conclusion

As the first independent empirical evaluation of the IPF method, our pilot study results suggest that this approach is promising for the treatment of CPTSD involving childhood trauma. This relatively novel psychotherapeutic technique brings something unique to the table: the possibility of improving representational models of attachment in adults using visualizations. We hope that more clinicians get involved in clinically investigating IPF’s potential, and that more research is performed to uncover its psychological underpinnings.

## Notes

1. The SIDES-SR scoring manual (Spinazzola, 2011) reports results from a validation study conducted with a PTSD sample (*N* = 63). The mean symptoms severity score for said sample was found to be 33.62, with a standard deviation of 19.12. The manual does not define a clinical cut-off for severity of symptoms. In the absence of such cut-off, we decided to use 33.62 as our inclusion cut-off score. Our sample SIDES-SR severity of symptoms mean was *M* = 61.18, *SD* = 16.99, almost double that.

2. Nonparametric analyses were also performed using Wilcoxon Signed-Rank tests finding highly similar results. The results of parametric analyses are therefore reported.
